# Blood Sample Collection in Randomized Controlled Trials for Biomarker Discovery and Validation: Experience of the PREOPANC-2 Trial

**DOI:** 10.1245/s10434-025-16890-0

**Published:** 2025-02-05

**Authors:** Esther N. Dekker, Quisette P. Janssen, Jacob L. van Dam, Gaby J. Strijk, Eva M. M. Verkolf, Sridhar Kandala, Jasper Dumas, Amine Fellah, Eileen M. O’Reilly, Marc G. Besselink, Casper H. J. van Eijck, Marjolein Y. V. Homs, Geert-Jan van Tienhoven, Johanna W. Wilmink, Dana A. M. Mustafa, Bas Groot Koerkamp, for the Dutch Pancreatic Cancer Group, L. V. Beerepoot, M. L. van Bekkum, B. A. Bonsing, H. Bos, K. P. Bosscha, S. A. Bouwense, L. Brouwer-Hol, A. M. E. Bruynzeel, O. R. Busch, G. Cirkel, P. P. L. O. Coene, J. W. B. de Groot, B. C. M. Haberkorn, I. H. J. T. de Hingh, T. M. Karsten, G. Kazemier, M. B. van der Kolk, M. S. L. Liem, O. J. L. Loosveld, S. A. C. Luelmo, C. M. Luyer, J. S. D. Mieog, V. B. Nieuwenhuijs, J. J. M. E. Nuyttens, D. ten Oever, G. A. Patijn, H. C. van Santvoort, M. W. J. Stommel, M. M. Streppel, A. ten Tije, E. Versteijne, J. de Vos - Geelen, R. F. de Wilde

**Affiliations:** 1 Department of Surgery, Erasmus MC Cancer Institute, Rotterdam, The Netherlands; 2 Department of Medical Oncology, Erasmus MC Cancer Institute, Rotterdam, The Netherlands; 3 Department of Pathology, Tumor Immuno-Pathology Laboratory, Erasmus MC, Rotterdam, The Netherlands; 4 Department of Medicine, Memorial Sloan Kettering Cancer Center, New York, NY USA; 5 Cancer Center Amsterdam, Amsterdam UMC, Amsterdam, The Netherlands; 6 Department of Surgery, Amsterdam UMC, Location University of Amsterdam, Amsterdam, The Netherlands; 7 Department of Radiation Oncology, Amsterdam UMC, Location University of Amsterdam, Amsterdam, The Netherlands; 8 Department of Medical Oncology, Amsterdam UMC, Location University of Amsterdam, Amsterdam, The Netherlands

**Keywords:** Biomarkers, Personalized medicine, Randomized controlled trial, Pancreatic cancer

## Abstract

**Background:**

This study aimed to investigate the feasibility and yield of blood sample collection in an investigator-initiated nationwide randomized controlled trial (RCT).

**Methods:**

In the PREOPANC-2 trial, 375 patients with (borderline) resectable pancreatic cancer were randomly assigned to two neoadjuvant regiments in 19 centers in the Netherlands (2018–2021). Blood sample collection was scheduled at seven time points before, during, and after treatment. The primary outcome was the proportion of successfully collected blood samples at each scheduled time point.

**Results:**

Of the 375 randomized patients, 12 were excluded from blood sample collection before any treatment. From the remaining 363 patients, 1513 (87 %) of 1748 blood samples were collected, processed, mailed, and centrally stored. The blood samples were collected before treatment from 347 (96 %) of the 363 patients, after the first neoadjuvant cycle from 322 (94 %) of 343 patients, after neoadjuvant treatment (i.e., before surgery) from 260 (83 %) of 313 patients, and after surgery from 210 (77 %) of 271 patients. During the follow-up visits, blood samples were collected from 147 (82 %) of 179 patients 12 months after randomization and from 83 (77 %) of 108 patients after 24 months. A total of 220 samples (13 %) were missing. The most common causes for missing blood samples were scheduling oversights, unsuccessful blood draw attempts, and mailing failures (151 times, 69 %). Blood sample collection was canceled 69 times (31 %) due to COVID-19.

**Conclusion:**

Blood sample collection in the PREOPANC-2 trial had a yield of 96 % before treatment and an overall yield of 87 %. Collection of blood samples for biomarker studies is feasible in a nationwide RCT.

Biomarkers in tumor tissue and blood samples are increasingly investigated for personalized oncology.^1^ Personalized oncology involves biomarker-based tailored treatment to improve prognosis and minimize treatment-related toxicity.^2,3^ Biomarkers that guide treatment decisions are called “predictive” biomarkers. These biomarkers predict treatment response, whereas “prognostic” biomarkers inform only about prognosis irrespective of treatment.^4^ A predictive biomarker could be a conventional blood test (e.g., carbohydrate antigen 19-9 [CA19-9]), a genomic alteration measured in tissue, or circulating tumor DNA (ctDNA), or a gene expression signature.^5^ An illustrative example is the identification of *KRAS* mutation as a predictive biomarker in patients with metastatic colorectal cancer, signifying resistance to cetuximab.^6,7^ Biomarkers also can monitor treatment response. The advantage of liquid biomarkers is that they can be repeatedly assessed, requiring only a peripheral blood draw.^3^

The PREOPANC-2 trial was an investigator-initiated nationwide randomized controlled trial (RCT) that compared two neoadjuvant regimens for pancreatic cancer: FOLFIRINOX and gemcitabine with radiation. The overall survival curves were overlapping. Some patients, however, may benefit more from one regimen than others. Several cohort studies have identified promising biomarkers predicting treatment response to either FOLFIRINOX or gemcitabine-based treatment.^8–10^ These non-randomized studies, however, typically focused on response to one treatment instead of predicting response to both regimens.

An RCT has the advantage of randomly assigned treatment and is required to validate promising predictive biomarkers.^11^ A predictive biomarker should demonstrate heterogeneity of treatment effects based on the value of the biomarker.^12^ The requirement of RCTs to validate predictive biomarkers underscores the importance of collecting both tissue and liquid biopsies in RCTs in oncology.^13^ Moreover, RCTs offer a unique opportunity to collect tissue and blood samples within a prespecified patient cohort during standardized treatment and follow-up assessment.

The collection, processing, mailing, and storage of blood samples can be challenging, particularly when performing a nationwide RCT. Establishing agreements in advance to address potential obstacles preemptively is essential to minimize missed or inadequate blood samples.^14^ Any missed sample will decrease the power of a biomarker study. Few studies have investigated the yield of blood sample collection for a better understanding of the challenges associated with collecting liquid biopsies in a multicenter RCT.

This study aimed to investigate the feasibility and yield of blood sample collection in an investigator-initiated nationwide RCT for patients with pancreatic cancer.

## Material and Methods

### Study Design and Participants

The PREOPANC-2 trial was an investigator-initiated nationwide phase 3 RCT in 19 Dutch centers comparing neoadjuvant FOLFIRINOX followed by surgery and neoadjuvant gemcitabine-based chemoradiotherapy followed by surgery and adjuvant gemcitabine. Full details of the study design and methods have been published previously.^15^ Adult patients (age ≥18 years) who had histologically or cytologically confirmed resectable or borderline resectable pancreatic ductal adenocarcinoma (PDAC) without distant metastases were eligible for the study. The supplementary criteria included a World Health Organization (WHO) performance status of 0 or 1 and ability to undergo surgery and chemo(radio)therapy.

All the patients provided written informed consent for study participation, biomaterial collection, and analysis. The study followed Good Clinical Practice guidelines and the Declaration of Helsinki. The institutional review boards of all the participating centers approved the study protocol.

The patients were randomly assigned in a 1:1 ratio to arm A (neoadjuvant FOLFIRINOX followed by surgery) or arm B (neoadjuvant gemcitabine-based chemoradiotherapy followed by surgery and adjuvant gemcitabine). Treatment in arm A comprised eight cycles of neoadjuvant FOLFIRINOX followed by surgery without adjuvant treatment. Treatment in arm B comprised three cycles of neoadjuvant gemcitabine combined with radiotherapy in cycle two, followed by surgery and four cycles of adjuvant gemcitabine. To detect a hazard ratio (HR) of 0.70 with 80 % power, 252 events were needed and expected to be reached with the enrollment of 368 eligible patients.

### Blood Sample Collection

**Fig. 1:**
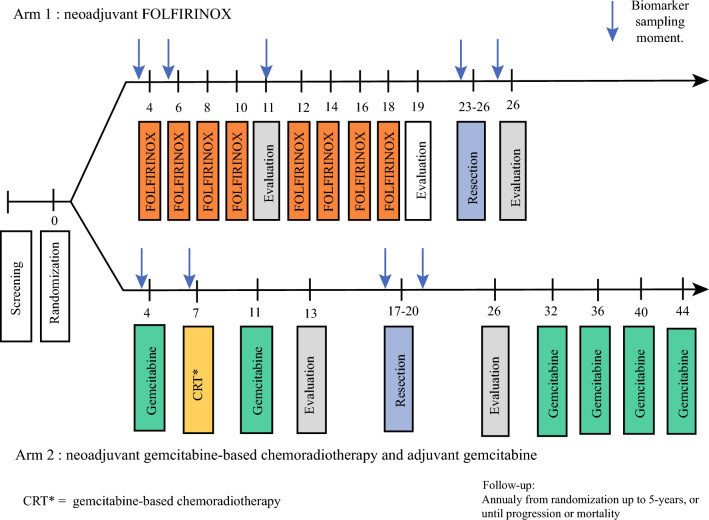
Schematic representation of the PREOPANC-2 trial with associated blood sampling moments from randomization until the end of treatment

Blood samples for biomarker measurements were collected in the two treatment arms at scheduled time points: before treatment, after cycle one, after four cycles of neoadjuvant FOLFIRINOX (only in arm A), before surgery, and before the start of adjuvant chemotherapy (within 45 days after resection). During follow-up visits, blood samples were collected annually until progression, until death, or until 5 years after randomization (Fig. 1).

The blood sample collection in the current study encompassed the period until the blood samples after 2 years of follow-up evaluation because the follow-up assessment of all the patients up to 2 years had been completed. The primary outcome was the proportion of successfully collected blood samples at each scheduled time point. The secondary outcomes were the leading causes of missed or inadequate blood samples.

The patients were eligible for a blood draw if they were alive, without disease progression, and still following the scheduled treatment. The patients were ineligible for a blood draw in cases of screening failure, clinical deterioration, or withdrawal of consent. Additionally, blood samples were marked as ineligible after cycles 1 and 4 when patients transitioned to surgery early but continued with the study protocol, which included scheduled blood draws before and after surgery. Blood samples were marked as missing if they were not collected despite the eligibility of the patient for a blood draw.

At each collection time point, peripheral venous blood samples were drawn in one 6-mL EDTA tube (Becton Dickinson, Franklin Lakes, NJ, USA), two 10-mL CellSave tubes (Menarini Silicon Biosystems, Castel Maggiore, Italy), one 10-mL serum tube (Becton Dickinson, Franklin Lakes, NJ, USA), and one 3-mL Tempus tube (Applied Biosystems, Foster City, CA, USA).

For the first 2 years of the trial, two CellSave tubes were collected, except in one center (Erasmus MC), which collected two 10-mL EDTA tubes instead of CellSave tubes. Since September 2020, all centers have transitioned to collecting three CellSave tubes.

The 6-mL EDTA tubes were used to collect tumor-educated platelets (TEPs). The CellSave and 10-mL EDTA tubes were used to collect plasma to isolate ctDNA and peripheral blood mononuclear cells (PBMCs). Serum tubes can be used to isolate serum samples. Tempus tubes, in which 3 mL of whole blood is stabilized with 6 mL of RNA stabilizing reagent, can be used to obtain total RNA from whole-blood samples.

### Blood Sample Labeling and Mailing

Each participant received a general study number and a unique biomarker identifier for every visit to encode personal information. The unique biomarker identifier consisted of a number that was specific for a hospital, followed by “PP2” and the patient number assigned within the trial for that particular hospital. For example, the first patient randomized at Erasmus MC received the label “001PP20001,” the second patient received the label “001PP20002,” and so forth. This approach enabled the transmission of labels containing the hospital code to respective hospitals, where staff could manually inscribe the patient's unique number onto the label.

The Erasmus MC (sponsor) supplied all the tubes and packaging material, including sealed envelopes with absorbing material, transport blisters, and safety bags, to the participating centers. After the blood collection, the tubes were labeled containing the biomarker identifier of the patient and the time of collection. Thereafter, the tubes were packed in a transport blister and safety bag. The CellSave, Tempus, and EDTA tubes collected in all 19 participating centers were sent to a central laboratory at Erasmus MC to be processed and stored. If the sample collection was on a Friday, the weekend, or a holiday, fast delivery mail was used to ensure receiving and processing of blood samples within 72 h after the blood draw. Because serum samples should be separated within 4 h after the blood draw, these blood samples were processed and stored in each participating center and mailed to the Erasmus MC every few months. The distance between Erasmus MC and the nearest participating center was 5 miles, whereas the farthest participating center was 155 miles away.

### Blood Sample Processing

The CellSave tubes were processed as soon as possible, within a maximum of 72 h after the blood draw, to obtain plasma, PBMCs, and the 6-mL EDTA tubes to obtain TEPs. The two 10-mL EDTA tubes collected within the Erasmus MC (instead of the CellSave tubes) were processed within 4 h to obtain plasma and PBMCs. The tubes were centrifuged at 1000 g for 10 min and again at 12,000 g for 10 min after transfer into new tubes to separate the plasma.

**Fig. 2:**
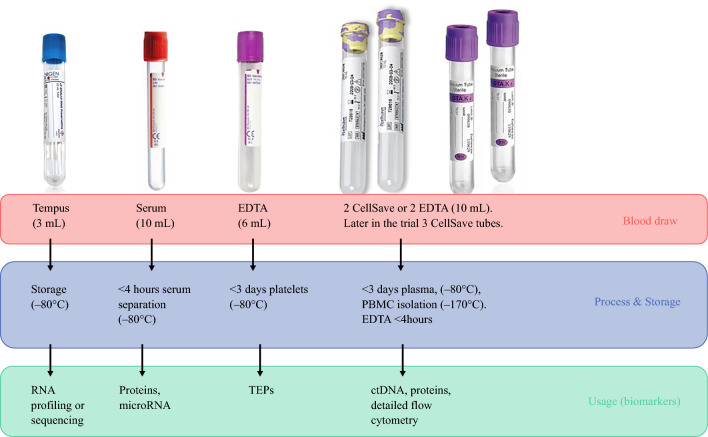
Blood sample tubes collected in the PREOPANC-2 trial

To extract PBMCs, the Ficoll-Paque technique was applied using LeucoSep tubes (Greiner Bio-One, Kremsmünster, Austria). The serum tubes were centrifuged for 10 min at 1000 g for serum extraction. For the extraction of TEPs, the 6-mL EDTA tubes were centrifuged for 20 min at 120 g and again for 20 min at 360 g after supernatant had been transferred into new 15-mL tubes. After centrifugation, plasma, serum, and TEP samples were aliquoted in 1.8-mL Nunc tubes and stored at –80 °C. The isolated PBMCs were aliquoted in 1.8-mL Nunc tubes, then frozen using a Mr. Frosty freezing container, and finally stored in liquid nitrogen until needed for further use. Because the Tempus tubes require no processing, they were labeled and immediately stored at –80 °C upon receipt (Fig. 2).

The sample database was adjusted immediately upon the inclusion of new blood samples. For every blood sample collection, the number of stored Nunc tubes and the yield of PBMCs were noted. Additionally, specific storage locations (e.g., –80 °C freezer, fourth floor, canister M, box 4, row 8, position 3; 4-8-3) were meticulously annotated to ensure efficient retrieval of samples when required.

## Results

In the PREOPANC-2 trial, 375 patients were randomized to neoadjuvant FOLFIRINOX (*n* = 188) followed by surgery versus neoadjuvant gemcitabine-based chemoradiotherapy followed by surgery and adjuvant gemcitabine (*n* = 187). Of the 375 patients enrolled in the trial, 12 (3.2 %) were excluded from blood sample collection before the start of treatment due to screening failures (*n* = 2), withdrawal of consent (*n* = 2), disease progression (*n* = 5), clinical deterioration (*n* = 2), or COVID-19 (*n* = 1).

**Fig. 3:**
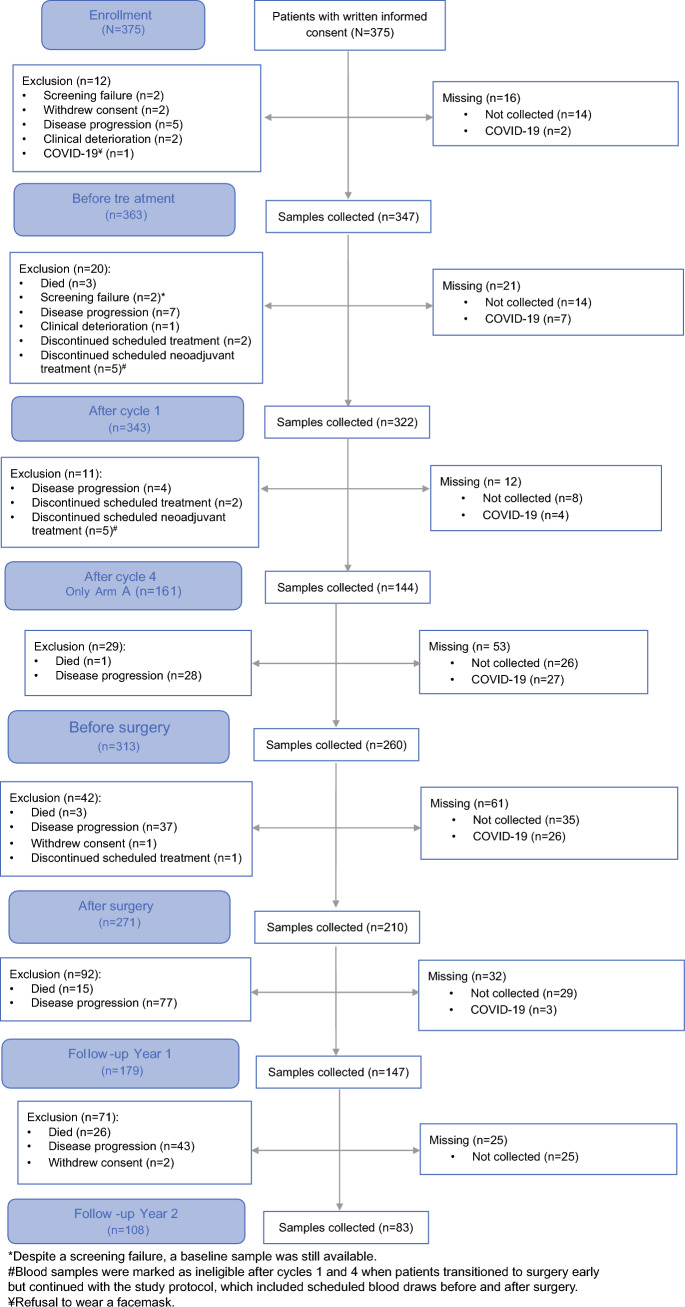
Flowchart of blood sample collection

During the trial, 1513 blood samples were successfully collected, mailed, processed, and stored for 363 patients from 19 centers. Each blood sample was collected at one of seven time points (Table 1). The theoretical maximum number of blood samples was 2625, with no accounting for mortality during the trial. The actual maximum number of blood samples for the patients eligible at the time of sampling was 1738. Therefore, the proportion of successful blood sample collection was 87.1 % (1513 of 1738), with 220 blood samples missing (12.7 %). Blood samples were canceled due to COVID-19 a total of 69 times (31.4 %) (Fig. 3). The remaining 151 missing blood draws (68.6 % of all missing draws) were attributed to potentially avoidable reasons including scheduling failures, sample acquisition failures, and mailing failures.

**Table 1:** Number and timing of blood sample collection in the PREOPANC-2 trial

	Before treatment	After cycle 1	After cycle 4 (only arm A)	Before surgery	After surgery	Year 1 follow-up	Year 2 follow-up	Total
Number collected	347	322	144	260	210	147	83	1,513
Patients eligible for blood draw	363	343	161	313	271	179	108	1,738
% Drawn of patients eligible for blood draw	95.6 %	93.9 %	89.4 %	83.1 %	77.5 %	82.1 %	76.9 %	87.1 %
% Drawn of all included patients	92.5 %	85.6 %	76.6 %	69.3 %	56.0 %	39.2 %	22.1 %	57.6 %

Before treatment, 363 patients were eligible for blood draws, and blood was successfully collected from 347 (95.6 %) of these patients. For 16 (4 %) of the 363 eligible patients, the blood draw before treatment was missed due to scheduling oversights or unsuccessful blood draw attempts (*n* = 14) (shown as “not collected” in the figure), or it was canceled because of COVID-19 (*n* = 2).

After the first cycle of neoadjuvant treatment, 322 (93.9 %) of 343 eligible patients underwent a blood draw. After four cycles of FOLFIRINOX, an extra blood draw was scheduled in arm A, with blood samples collected from 144 (89.4 %) of 161 patients alive in arm A. Before surgery, 260 (83.1 %) of 313 patients alive underwent a blood draw. After surgery, 210 (77.5 %) of 271 patients alive underwent a blood draw.

During the first follow-up visit 1 year after randomization, 147 (82.1 %) of 179 patients alive underwent a blood draw. Finally, 2 years after randomization, 83 (76.9 %) of 108 patients alive underwent a blood draw. The proportion of blood draws for all the eligible patients decreased over time, from 95.6 % before treatment to 76.9 % after 2 years of follow-up evaluation (*p* = 0.21).

## Discussion

In the PREOPANC-2 trial, an investigator-initiated nationwide RCT, blood samples were collected at seven time points in 19 centers throughout the Netherlands. Altogether, 1513 (87 %) of 1738 potential blood samples were collected from 363 patients. Over time, 220 blood samples (13 %) were missed. Potentially avoidable reasons were scheduling failures, sample acquisition failures, and mailing failures at 151 time points (69 %). Moreover, 69 (31 %) of the missing samples were canceled due to the COVID-19 pandemic during the trial.

The American Society of Clinical Oncology (ASCO) has emphasized the need for trial sponsors to establish a comprehensive biospecimen bank for each clinical trial to promote biomarker-driven research.^16^ Moreover, the National Comprehensive Cancer Network (NCCN) guideline recommends the preservation of tumor tissue, together with matched blood and serum samples, for patients participating in clinical trials for pancreatic cancer.^13^ These recommendations underscore the awareness that RCTs are the perfect opportunity to discover and validate predictive biomarkers.

Based on the published literature, however, we concluded that the collection of blood samples has not been accomplished in RCTs for pancreatic cancer that inform the current standard of care. For example, although the study protocols of the ESPAC-4^17^ and PRODIGE-24^18^ trials indicated an intention to collect blood samples for translational research, no publication on this collection has emerged to date. In addition, to our knowledge, the ESPAC-3^19^ and PRODIGE-4^20^ trials also have not reported on blood sample collection. This lack of blood sample collection or publication in these key RCTs can be attributed to the inherent complexity of the blood sample collection process. It requires an excellent infrastructure for scheduling, processing, mailing, and storage of blood samples. Blood sample collection can be successful only with the commitment of patients, the multidisciplinary team, local research nurses, and the trial’s coordinating team. The ongoing PASS-01 trial (NCT04469556) randomizes patients with metastatic PDAC to FOLFIRINOX versus gemcitabine with nab-paclitaxel. This RCT is unique because one of its primary aims is to identify predictive biomarkers.^21^

The costs of all the necessary steps to biobank a single blood sample accumulate to a price between 100 and 1000 euros. For the current study, that would add up to between 150 thousand and 1.5 million euros. It can be challenging for investigator-initiated studies to obtain funding for blood sample collection. Blood samples are helpful for biomarker studies only when accrual and follow-up evaluation have been completed, which can easily take 10 years from the application for funding to the completion of follow-up evaluation. The most promising biomarkers will most likely have changed during that period. We recommend that funding agencies should require and consider funding blood sample collection in RCTs. The decision about which biomarkers to study, however, should be determined on the basis of the available literature at the time when accrual and follow-up evalution are completed.

For other cancers, several RCTs have collected blood samples. In the S0500 RCT for metastatic breast cancer, blood samples were collected before the start of treatment and on day 22 for the assessment of circulating tumor cells (CTCs). This study successfully collected blood samples from 595 (95 %) of 624 enrolled patients before the start of treatment and for 288 (90 %) of 319 patients on day 22.^22^ The CALGB/SWOG 80702 (Alliance) RCT randomized 2526 patients with stage III colon cancer to receive celecoxib or placebo, both combined with adjuvant chemotherapy. Before the start of treatment, 1900 blood samples (75 %) were collected in 654 centers throughout the United States and Canada.^23^ The yield of blood sample collection in these RCTs was very high, similar to the current study. The authors did not report the causes of missed blood samples or discuss potentially avoidable causes with future recommendations.

Blood sample collection in RCTs is essential for biomarker discovery and validation of promising circulating biomarkers for personalized oncology. Heterogeneity in treatment effect (e.g., of targeted treatments) can be expected based on *in vivo* and animal experiments or cohort studies but requires confirmation in RCTs. In the study by Lièvre et al.,^6^ the relative risk of responding to cetuximab was 10-fold higher for the patients who had metastatic colorectal cancer without KRAS mutations than for those with KRAS mutations (HR, 10.5; 95 % confidence interval [CI], 2.1–51.1). The authors concluded that a prospective randomized study was needed for validation.

The EURTAC trial is an example of an RCT that substantiated the clinical evidence confirming the presence of EGFR tyrosine kinase as a predictive biomarker for erlotinib in patients with advanced non-small cell lung cancer (NSCLC).^24^ This trial demonstrated that erlotinib was superior to the standard chemotherapy as first-line therapy for advanced NSCLC with EGFR activating mutations (HR, 0.37; 95 % CI, 0.25–0.54; *p* < 0.01).^24^ After the findings of the EURTAC trial and subsequent studies conducted in Asian populations, anti-EGFR tyrosine kinase inhibitors have become the standard of care for patients with EGFR mutation-positive NSCLC, as recommended by the NCCN guideline.^25^

The collection of blood samples has posed challenges in other RCTs. Coleman et al.^14^ requested a code of practice to protect the samples donated by trial participants. They brought attention to the D-CARE trial, a placebo-controlled phase 3 RCT of adjuvant denosumab in 4509 women with stage II or III breast cancer, sponsored by a pharmaceutical company. More than 80,000 biologic samples were never used in biomarker studies and discarded.

The PREOPANC-2 trial found similar overall survival when comparing neoadjuvant FOLFIRINOX with gemcitabine-based radiation.^26^ Several promising biomarkers to predict the treatment effect of gemcitabine or FOLFIRINOX have been identified using a pretreatment biopsy or blood sample.^8,10,11,27–31^ Such baseline blood samples were collected from 93 % of all the randomized patients in the PREOPANC-2 trial. Biomarker studies using blood samples after one or four cycles of systemic chemotherapy potentially may avoid continuation of ineffective treatments. Immediately before surgery, a blood sample-based biomarker may identify patients with predicted poor survival after surgery, potentially leading to a recommendation to withhold surgery.^32^ A blood sample immediately after surgery could guide adjuvant treatment.^33,34^ Finally, blood samples during follow-up evaluation could detect or predict early disease recurrence.^35,36^

Unfortunately, RCTs are typically underpowered to evaluate biomarkers because they are powered to detect a clinically relevant difference between two treatment arms. A dichotomous predictive biomarker (e.g., ctDNA low or high) would expand the comparison to four groups, each treatment arm with or without the biomarker. Consequently, the RCT is powered only to validate a strong predictive biomarker. The power further decreases for blood samples obtained during and after treatment, because patients die. The number of patients alive to obtain blood samples went from 363 before treatment to 108 after 2 years of follow-up evaluation. Moreover, in the current study, the proportion of collected blood samples decreased from 96 % before treatment to 77 % after 2 years of follow-up evaluation.

With the PREOPANC-2 trial, we demonstrated the feasibility of blood sample collection in an investigator-initiated nationwide RCT, with an overall collection yield of 87 %. To achieve this success, certain conditions had to be met. Effective collaboration between the sponsor and participating centers was facilitated through study initiation visits conducted by the study coordinator at each center. The presence of a research nurse alongside a local principal investigator (PI) at each center proved crucial. The local PIs were pivotal for patient identification and inclusion, patient oversight, and task delegation. The research nurses maintained constant communication with the Erasmus MC study team, ensuring the acquisition of necessary tubes and packaging materials and coordinating blood draws, processing, and mailing. Blood draws were scheduled mostly during already planned outpatient clinic visits.

Building on the literature and our own experiences, we propose several recommendations for blood sample collection in RCTs. Before trial initiation, researchers should carefully consider the type and number of tubes.^37^ This can be challenging because what seems best when the protocol is written may differ 10 years later when the follow-up evaluation is completed and all blood samples are ready for biomarker studies. In the PREOPANC-2 trial, we changed from EDTA to Cellsave tubes during the trial, which should be avoided. Furthermore, standard operating procedures are required, from scheduling of the blood draw, to storage to ensure uniformity.

In addition, researchers should determine whether blood samples should be processed centrally or locally based on the estimated time between blood collection and processing. Given the reliability of the mailing system in the Netherlands and the relatively short distances within the country, it was feasible to transport blood samples by mail. However, this approach may be less reliable in larger countries. This challenge can be addressed by processing and storing blood samples at the hospital where the blood draw is performed. These samples then can be shipped together every 6 months.

Finally, a dedicated study team in each hospital, including a research nurse, should be established, and this team will be responsible for coordinating and facilitating blood draws for blood sample collection.

In addition to the blood samples, we also have collected the initial tumor biopsies and resected specimens of all patients. We are currently performing a systematic review to identify the most promising predictive biomarkers for the treatment effect of FOLFIRINOX and/or gemcitabine-based treatment for patients with pancreatic cancer. Recently, several promising biomarkers have been identified.^8,9,29,30^ In the next few years, we aim to validate the most promising biomarkers with an international team of experts in pancreatic cancer, translational research, and bioinformatics.

The current study has several limitations. First, our database does not allow us to differentiate whether a blood sample was not collected due to scheduling, sample acquisition, or mailing failure. However, we assume that most of these missed blood samples can be attributed to a failure of scheduling because a failed blood draw is rare and the Dutch mailing services are reliable.

Second, most collected blood samples have not yet been used for biomarker analysis. Consequently, we could not identify the number of samples that cannot be used due to poor quality.

In conclusion, we demonstrated that blood sample collection was feasible in an investigator-initiated nationwide RCT with an overall yield of 87 %. The reasons for missed blood draws were scheduling and blood draw failures (69%). Moreover, 31 % of the missing samples were due to COVID-19.
